# Insights into the cotton anther development through association analysis of transcriptomic and small RNA sequencing

**DOI:** 10.1186/s12870-018-1376-4

**Published:** 2018-08-03

**Authors:** Jin Chen, Pin Su, Pengyun Chen, Qiong Li, Xiaoling Yuan, Zhi Liu

**Affiliations:** 1 College of Bioscience and Biotechnology, Hunan Agricultural University, Changsha, 410128 China; 2 Hunan Academy of Agricultural Sciences, Institute of Plant Protection, Changsha, 410125 China; 3 State Key Laboratory of Cotton Biology, Institute of Cotton Research of CAAS, Anyang, 455000 China

**Keywords:** Cotton, Transcriptome, microRNA, Anther development, Gene expression regulation

## Abstract

**Background:**

Plant anther development is a systematic and complex process precisely controlled by genes. Regulation genes and their regulatory mechanisms for this process remain elusive. In contrast to numerous researches on anther development with respect to mRNAs or miRNAs in many crops, the association analysis combining both omics has not been reported on cotton anther.

**Results:**

In this study, the molecular mechanism of cotton anther development was investigated with the employment of association analysis of transcriptome and small RNA sequencing during the predefined four stages of cotton anther development, sporogenuous cell proliferation (SCP), meiotic phase (MP), microspore release period (MRP) and pollen maturity (PM). Analysis revealed that the differentially expressed genes are increasingly recruited along with the developmental progress. Expression of functional genes differed significantly among developmental stages. The genes related with cell cycle, progesterone-mediated oocyte maturation, and meiosis are predominantly expressed at the early stage of anther development (SCP and MP), and the expression of genes involved in energy metabolism, flavonoid biosynthesis, axon guidance and phospholipase D signaling pathways is mainly enriched at the late stage of anther development (MRP and PM). Analysis of expression patterns revealed that there was the largest number of differentially expressed genes in the MP and the expression profiles of differentially expressed genes were significantly increased, which implied the importance of MP in the entire anther development cycle. In addition, prediction and analysis of miRNA targeted genes suggested that miRNAs play important roles in anther development. The miRNAs ghr-miR393, Dt_chr12_6065 and At_chr9_3080 participated in cell cycle, carbohydrate metabolism and auxin anabolism through the target genes, respectively, to achieve the regulation of anther development.

**Conclusions:**

Through the association analysis of mRNA and miRNA, our work gives a better understanding of the preferentially expressed genes and regulation in different developmental stages of cotton anther and the importance of meiotic phase, and also the involvement of miRNAs in precise regulation for this process, which would be valuable for clarifying the mechanism of plant anther development in response to internal and external environments.

**Electronic supplementary material:**

The online version of this article (10.1186/s12870-018-1376-4) contains supplementary material, which is available to authorized users.

## Background

Cotton (*Gossypium hirsutum* L*.*) is one of the most economically important crops due to its fiber used as the principal natural source for the textile industry worldwide. The development of flowers directly determines the formation of cotton yield. So far, studies on male gametophytes have focused on model plants, such as *Arabidopsis*, [1–3], but there are less research reports on cotton. Though the basic mechanisms of anther development could be cross-referenced, each species has its own peculiarity [4]. The occurrence of flowers to the maturity of pollen grains is a systematic and complex process involving the regulation of a large number of genes [5–7]. Reportedly, genes involving in anther development concentrated on regulation of starch and sucrose metabolism, carbohydrate metabolism, antioxidant production, flavonoid biosynthesis, cell cycle, meiosis and plant hormone pathways [4, 8, 9]. In early stages of pollen development, large amounts of sugars are directed to the anther to support its development [10]. At late stage of pollen development and maturation, starch is required as energy storage which contributes to the secondary cell wall thickening and the swelling of the pollen grains, and ultimately promotes the dehiscent of the anther [5, 11]. Therefore, the disturbances in sugar and starch synthesis, transportation and metabolism during this stage could severely impair pollen development, resulting in male sterility [12]. Compared with the wild type, male sterile mutant shows declination in starch and sucrose production [8, 13]. Further investigations revealed that genes, including *Ghck1*, *SnRK1*, hexokinase and 14–3-3 protein genes are involved in the metabolism of sucrose in starch biosynthesis. Differential expression of these genes can lead to male sterility [9, 13–15]. Mitochondria, chloroplasts and peroxisomes are of great importance for energy-related metabolism, and also the site of reactive oxygen species (ROS) production. Pollen and tapetum cells are known to harbor a great number of mitochondria, and exhibit high respiration during pollen development [16]. Flavonoids are free radical scavengers and also are components of pollen coat [17]. During anther development of upland cotton, many genes related to the biosynthesis of quercetin, kaenpferol and myricetin were found with higher expression than in other tissues [4]. Plant hormones are important metabolic regulator, and the occurrence of male sterility in plants is usually accompanied by changes in the content of endogenous hormones in various reproductive organs [18]. GA is an absolute requirement for flower initiation [19], which involved in the development of tapetal and programmed cell death [20]. The synthesis and accumulation of GA in anthers is mainly regulated by the *GA20ox* and *GA3ox1* [21, 22]. The differential expression of *GA20ox* and *GA3ox1* usually leads to the occurrence of male sterility [13, 22]. IAA is another essential plant hormone that regulates plant growth and development [23]. Low content of IAA in anthers is the main reason of male sterility, and the low expression of *ILR1* and indole-3-acetic acid-amido consequently lead to low levels of IAA [13]. Similarly, the high level of IAA is also detrimental to anther development, which can increase the indehiscent anther ratio [9]. MicroRNAs (miRNAs) are a class of endogenous small regulatory non-coding RNA which negatively regulates expression of target genes at the post-transcriptional through degrading target mRNAs or repressing gene translation [24]. Resent researches showed that miRNAs were involved in multiple developmental processes, including seed germination [25], root development [26] and floral organ identity [27]. Studies showed that miR159 participated in the development of male gametophytes through target genes *DUO1* [28], and overexpression of miR159 lead to male sterility [29]. The genes *ARFs* play important roles in floral development [30], overexpression of targeted miRNA160 in cotton can induce anther indehiscence [31].

So far, a few studies on the molecular mechanism of anther development were carried out through separated applications of miRNAs and transcriptomes [32, 33], yet the association analysis combining both omics has not been reported. In our study, the association analysis of mRNA and miRNA in cotton anther at four continuous development stages was carried out, and a better understanding of the genes expression and regulation for this process was achieved.

## Results

### DEGs between different stages during anther development

Based on the statistical analysis of sequencing results, the 6.8 Gb raw bases data can be measured for each sample on average, and the ratio of clean bases is above 95%. The comparative analysis of cotton reference genome showed that the average mapped ratio of clean reads is 81.5% (Additional file 1: Table S1). The distance between samples is calculated by the clustering method, and the results showed that the two biological repetitions samples which cluster together preferentially at different stages (Additional file 2: Figure S1).

**Fig. 1:**
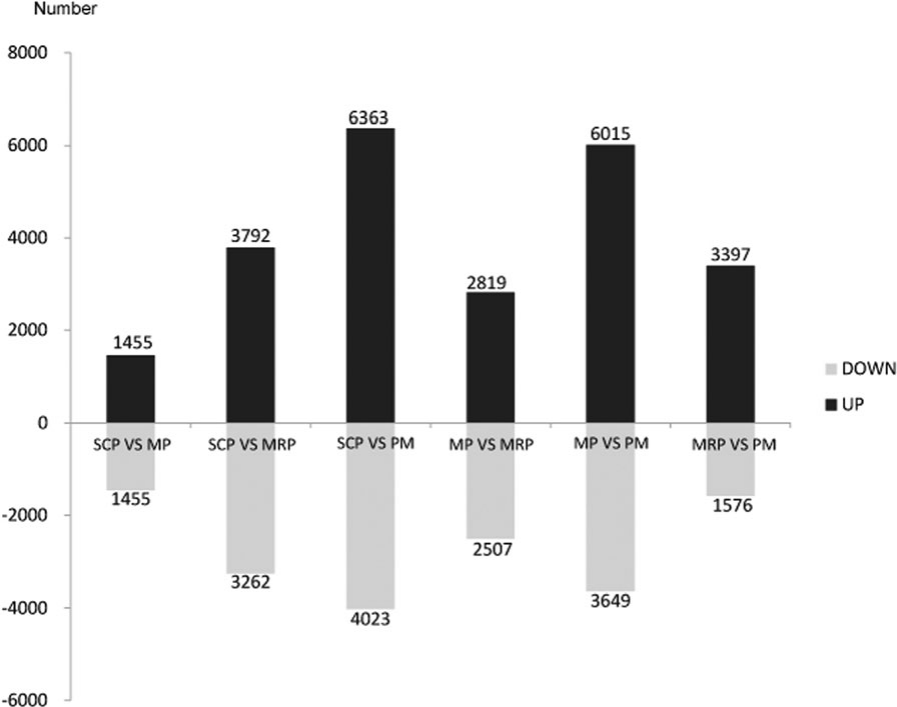
Histograms of DEGs with different control. The first three groups of bar charts were the number of DEGs obtained using SCP as control; the fourth and fifth groups of bar charts were based on MP as the control, and they were compared with MRP and PM respectively; the last of bar chart is based on MRP as control and compared with PM

In order to characterize the gene expression in anther development, the differentially expressed genes (DEGs) were screened between the four continuous stages, and 2910, 7054 and 10,386 DEGs were identified when SCP compared with MP, MRP and PM, respectively; When MP compared with the MRP and PM, 5326 and 9664 DEGs were screened respectively, and 4973 DEGs were obtained from the comparison between MRP and PM (Fig. 1). The results indicate that the number of DEGs is gradually increased with the progress of anther development, suggesting that more specific genes are recruited at different developmental stages to ensure the normal development and maturation.

### Functional analysis of DEGs

**Fig. 2:**
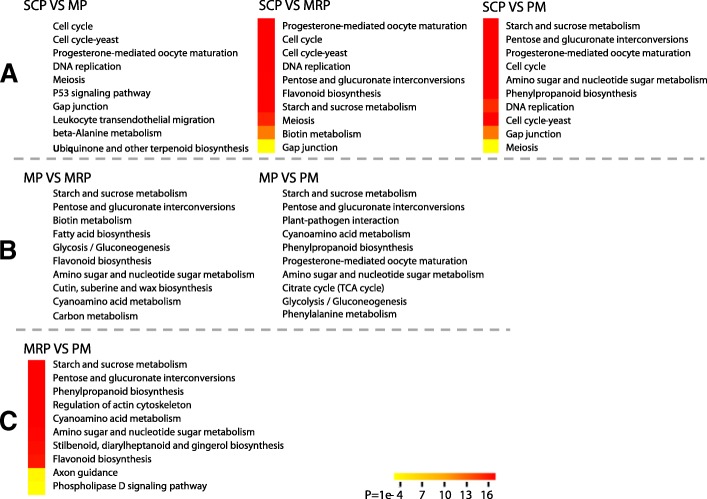
Heat maps of the KEGG pathway enrichment statistics. **a** Analysis of differentially expressed genes with KEGG enrichment using SCP as control; **b** Analysis of differentially expressed genes with KEGG enrichment using MP as control; **c** Analysis of differentially expressed genes with KEGG enrichment using MRP as control; *P*-values range from 0 to 1, and its less value means greater intensiveness. Top 10 pathway terms enriched by KEGG database are dispalied

To further understand the function of DEGs, we performed Kyoto Encyclopedia of Genes and Genomes (KEGG) enrichment analysis. The DEGs which were screened out with SCP as a control and compared with MP, MRP and PM, respectively, were mainly enriched in cell cycle, progesterone-mediated oocyte maturation and meiosis pathways (Fig. 2a and Additional file 3: Table S2). These pathways were necessary for early stage of anther development. The vast majority of DEGs which are obtained from MP compared with MRP and PM were involved in energy metabolic pathways, such as starch and sucrose metabolism, pentose and glucuronate interconversions and glycolysis/gluconeogenesis pathways (Fig. 2b and Additional file 3: Table S2); The DEGs between MRP and PM were mainly enriched in energy metabolic pathways, in addition to the flavonoid biosynthesis, axon guidance and phospholipase D signaling pathways (Fig. 2c and Additional file 3: Table S2).

**Fig. 3:**
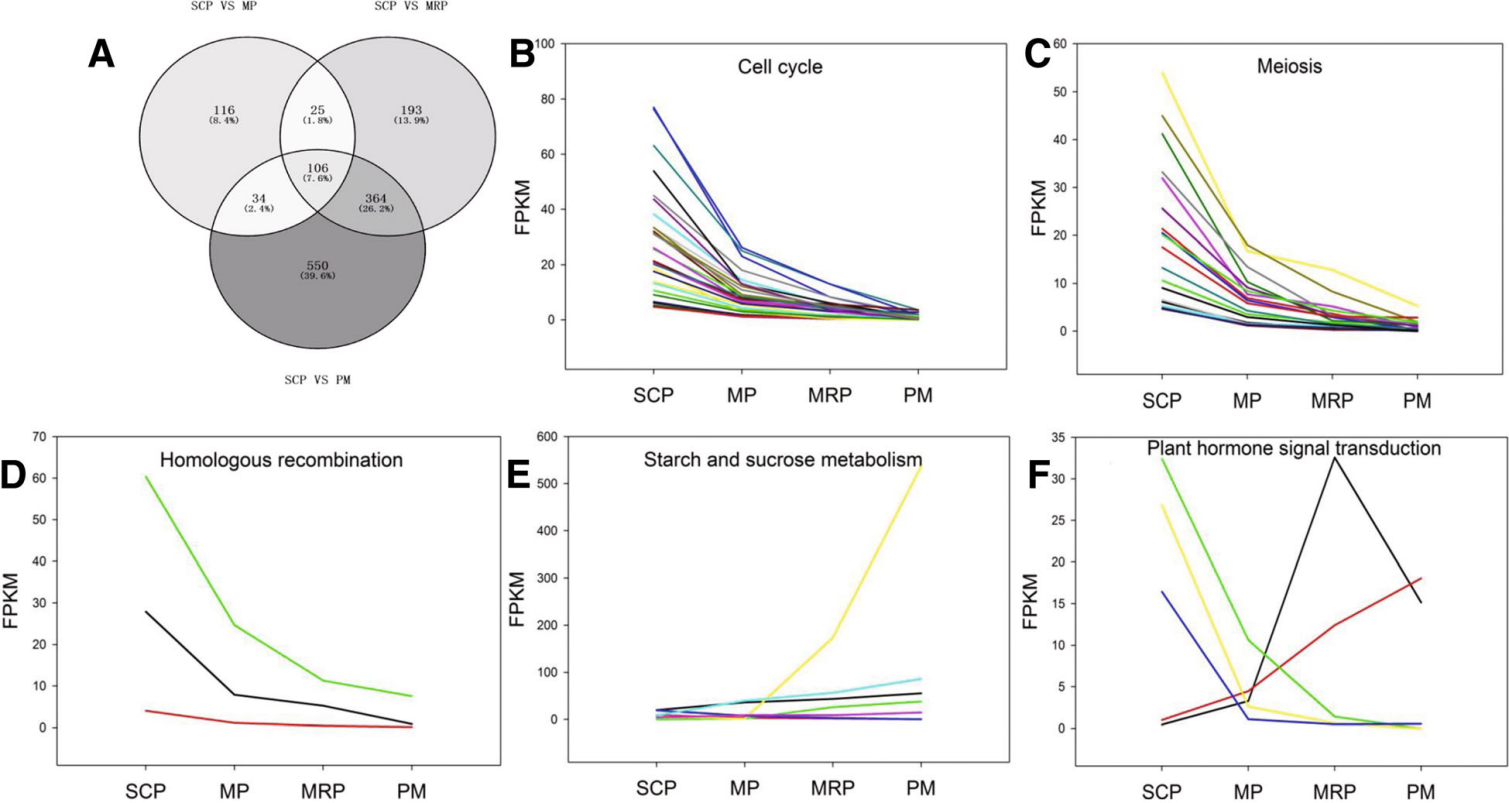
Expression profiles of genes related to metabolism and signaling pathways for anther development. **a** Venn diagram of anther development-related genes in three differentially expressed genes obtained from SCP as control; **b**-**d** The number of differential genes enriched in the cell cycle, meiosis and homologous recombination pathways, respectively; **e** Starch and sucrose metabolism was enriched in seven differentially genes; **f** Plant hormone signal transduction pathway was enriched five differential genes, and different hormone synthesis related genes showed different expression trends. The horizontal axis represents stages, and the vertical axis shows the stages series of gene expression for the gene after Log normalized transformation

About 29 metabolic and signal pathways, which related with the development of pollen and anther [4, 34], such as cell cycle, meiosis, glutathione metabolism and homologous recombination were selected for further enrichment analysis. Among the DEGs which came from individual comparison between SCP as a control and other three stages, and 281, 688 and 1054 DEGs were enriched respectively in the above-mentioned 29 pathways. A total of 106 genes were shared by three sets of DEGs (Fig. 3a and Additional file 4: Table S3) and These genes were clustered into the eleven pathways, such as cell cycle, meiosis, starch and sucrose metabolism and so on. The pathway which enriched the largest number of DEGs is cell cycle, followed by the meiosis with 30 and 18 DEGs, respectively. This suggested the importance of cell cycle and meiosis during the whole anther development process [1]. Among the 106 DEGs, the expression profiles of genes enriched in pathways like cell cycle, homologous recombination, meiosis, flavonoid biosynthesis and peroxisome exhibited downward trend with the development of anther (Fig. 3b, c and d; Additional file 4: Table S3; Additional file 5: Figure S2). While the genes participated in carbohydrate digestion and absorption and starch and sucrose metabolism showed the upward expression trend continuously (Fig. 3e; Additional file 4: Table S3; Additional file 5: Figure S2), which serves as a marker of pollen maturity and provides an energy reserve for pollen germination [9, 11]. The genes involved in carbon metabolism and plant hormone signal transduction had no consistent expression trend (Fig. 3f and Additional file 4: Table S3).

### Expression pattern of DEGs varied during anther development

**Fig. 4:**
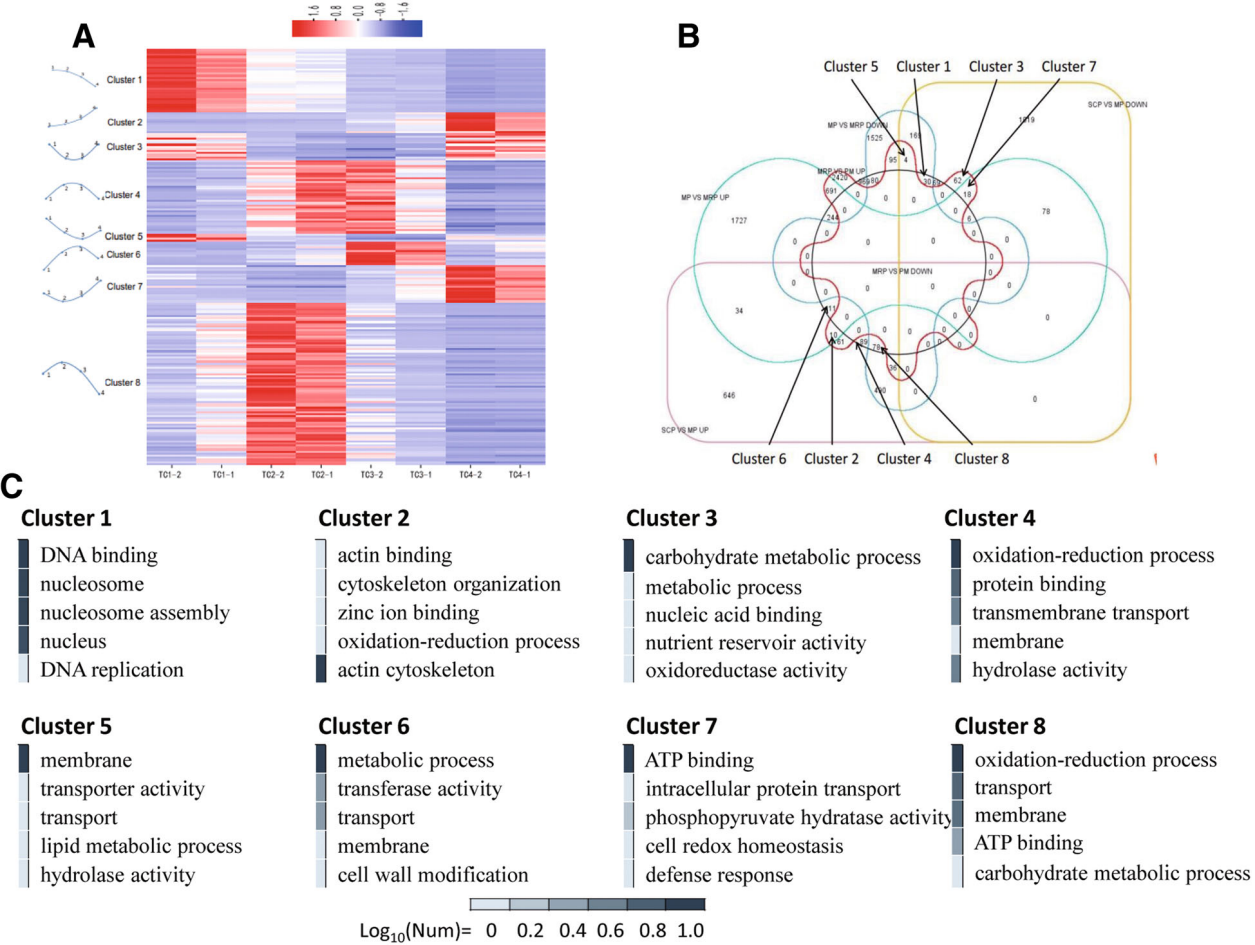
Gene expression patterns of the DEGs. **a** The heat map shows the K-means clustering of transcription levels of the DEGs. Genes were clustered into eight groups with distinct expression patterns in four stages. **b** DEGs Venn diagram. Eight groups expression patterns displayed on Venn diagram. **c** The top five most significantly enriched GO terms in each clusters; Log_10_ (Num) range from 0 to 1, and its larger value means greater

Three sets of DEGs are obtained with SCP as a control and compared with MP, MRP and PM, respectively, and eight main kinds of expression pattern are analyzed (Fig. 4a and b; Additional file 6: Table S4).Cluster 8 comprised the largest number of genes (78 genes), followed by cluster 4 (35) and cluster 1 (30). The expression profile of DEGs which contained in cluster 8 reached the highest in the MP (Fig. 4a and Additional file 6: Table S4), indicating that meiosis normally proceeded and required a large number of highly expressed genes [35]. Gene Ontology (GO) enrichment found that DEGs were mainly enriched in the oxidation-reduction process, transport, membrane, carbohydrate metabolic process and ATP binding terms (Fig. 4c).

In cluster 4, the genes expression reached the highest in the MP and MRP (Fig. 4a and Additional file 6: Table S4), which was consistent with the fact that MP require a large number of specific genes to participate. The GO enrichment results were mostly the same as cluster 8, and with part of DEGs enriched in the hydrolase activity and protein binding terms (Fig. 4c). The DEGs enriched in cluster 1 had the highest expression at the SCP, and then decreased with the process of anther development (Fig. 4a and Additional file 6: Table S4), GO annotation indicated that these genes related with DNA binding, nucleosome assembly and DNA replication were necessary for the proliferation of pollen mother cells (Fig. 4c). The remaining five clusters were enriched with a small number of differentially expressed genes and there was no significant GO enrichment.

### Different expression of miRNAs during anther development

By deeply small RNA sequencing anther samples at four stages, an average of 21.1 M clean reads was obtained for each sample. The obtained clean reads were aligned to the reference genome and the average alignment rate was found to be 93.86% (Additional file 7: Table S5). The clean reads were homologous alignment in the miRBasa, meanwhile, new miRNA predictions were performed for reads that failed to undergo homology comparisons. It was found that 97 miRNAs were detected from all samples, including 56 known miRNAs and 41 new miRNAs, respectively.

**Fig. 5:**
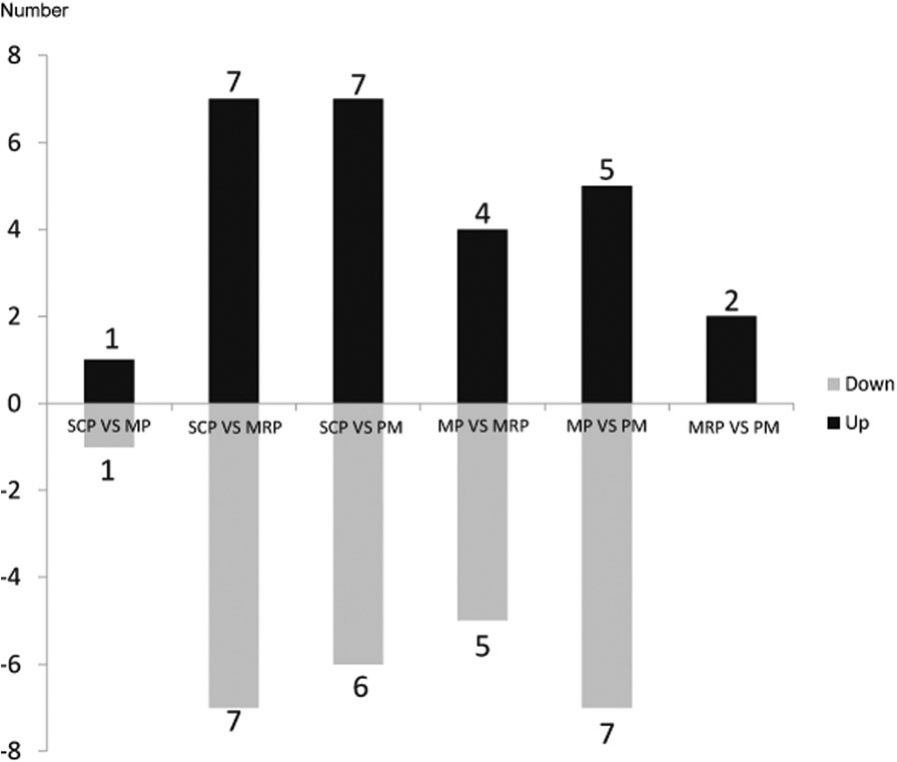
Histograms of differentially expressed miRNAs with different control. The first three groups of bar charts were the number of differentially expressed miRNAs obtained using SCP as control; the fourth and fifth groups of bar charts were based on MP as the control, and they were compared with MRP and PM respectively; the last of bar chart is based on MRP as control and compared with PM

When SCP compared with the MP, MRP and PM, respectively, 2, 14, and 13 differentially expressed miRNAs were obtained (Fig. 5). This trend was similar to the number of DEGs, presumably because more different genes were involved in the regulation of anther development, thus more miRNAs were required to regulate the mRNA expression [32]. When MP was used as a control and compared with the MRP and PM, 9 and 12 differentially expressed miRNAs were screened out (Fig. 5), respectively, and two difference expression miRNAs were obtained from the comparison between MRP and PM (Fig. 5).

### Target gene prediction of differentially expressed miRNAs

The target genes of different expression miRNAs were predicted in the DEGs of same phase of transcriptome sequencing and enrichment on anther development related pathways. In the comparison of SCP and MRP, there were a total of nine target genes which were predicted for seven miRNAs. In the comparation of SCP and PM, eight out of 13 differentially expressed miRNAs predicted 13 target genes in total (Additional file 8: Table S6; Additional file 9: Table S7). However, no target gene was predicted when comparing with SCP and MP.

KEGG enrichment analysis of the predicted target genes found that target genes were mainly enriched in pathways like cell cycle, glutathione metabolism and plant hormone signal transduction (Additional file 10: Figure S3; Additional file 11: Figure S4), suggesting the importance of corresponding miRNA in pollen development [30, 36]. GO clustering analysis found that target genes mainly clustered into molecular function (MF) and biological process (BP), and few genes in cell component (CC).

**Fig. 6:**
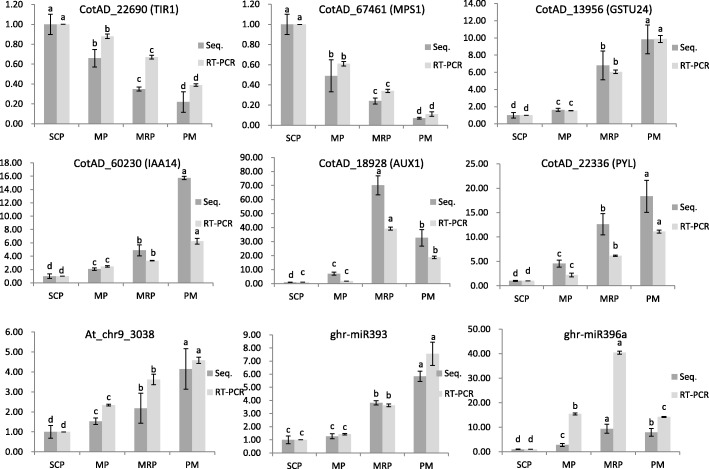
Gene expression analysis. qRT-PCR analysis of mRNAs (*TIR1, MPS1, GSTU24, IAA14, AUX1, PYL*) and miRNAs (At_chr9_3038, ghr-miR393, ghr-miR396a) expression at four developmental stages. Error bars represent SD, which were calculated from three technical repeats. Significant differences between treatments are indicated by letters above each bar (*P ≤ 0.05*). Data are present as means ± SE (*n* = 5)

**Fig. 7:**
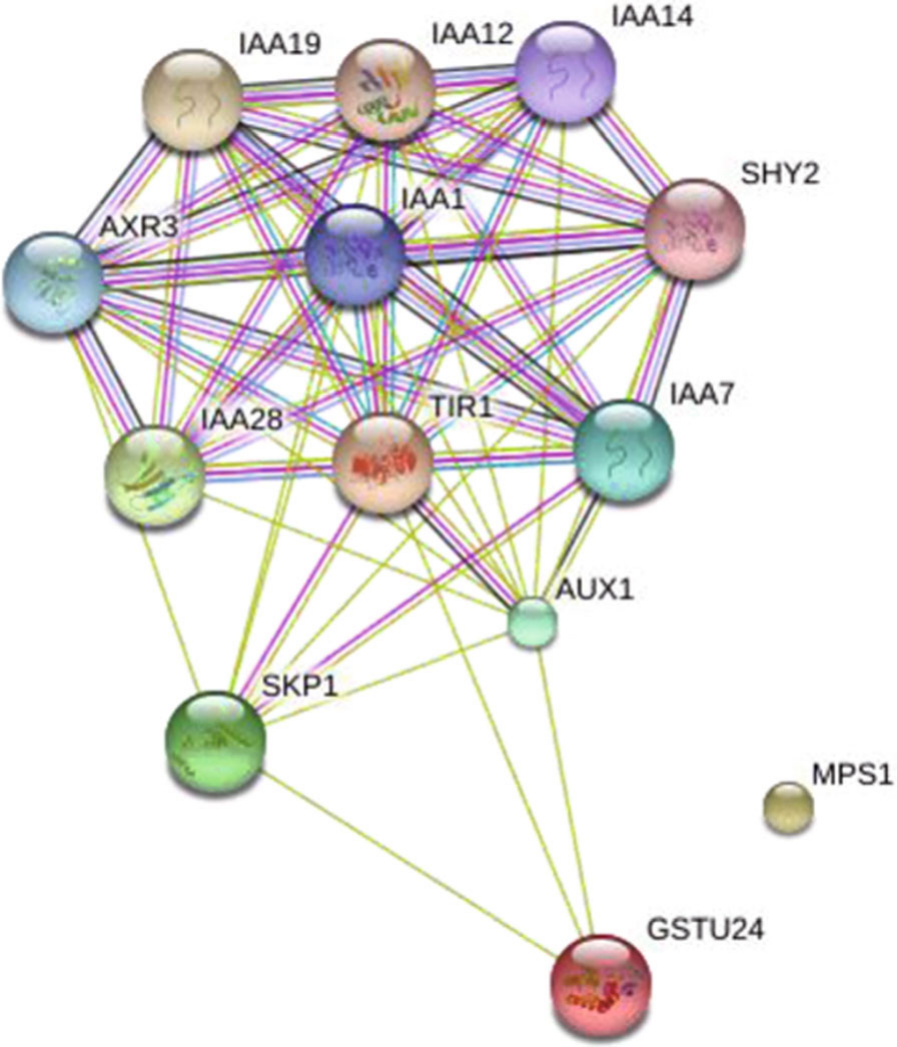
Protein-protein interaction (PPI) network. Differential miRNAs obtained with SCP as control group, three common target genes TIR1, MPS1 and GSTU24 PPI network illustrated by STRING (high confidence score = 0.7). Among the predicted genes, only IAA14 was significant differently expressed at different developmental stages

Among the predicted target genes, there were three sharing target genes, termed CotAD_22690 (*TIR1*), CotAD_67461 (*MPS1*) and CotAD_13956 (*GSTU24*), and the corresponding miRNAs were At_chr9_3038, ghr-miR393 and ghr-miR396a, respectively. The expressions analysis showed that all three miRNAs and *GSTU24* were up-regulated in the whole process of anther development, while the *TIR1* and *MPS1* were always down-regulated (Fig. 6). The interaction of these three target genes was predicted with string website (Fig. 7). As TIR1 is auxin receptor and mediates Aux/IAA proteins proteasomal degradation, it had been verified interaction with several IAAs, but its interaction with GSTU24 remained putative [37]. MPS1 had no interaction with the other two proteins.

The other genes were not found expressed in our transcriptome sequencing, but the *IAA14* was up-regulated. IAA14 functions as repressors of early auxin response at low auxin concentrations [38] and TIR1 functions as promoting the degradation of IAA14 [31, 39], which could explain the expression of *TIR1* decreased while the expression of *IAA14* increased during anther development.

## Discussion

### Metabolism and signal pathways in anther development

The comparative analyses among four stages showed that more DEGs existed between distant stages (Fig. 1), which indicate that more specific genes started to participate in the regulation with the process of anther development [40, 41].

KEGG enrichment analysis of the DEGs, which were obtained with the SCP as control (Fig. 2a), the results indicate that anthers made material reserves for the following meiosis and ensured the transition from SCP to MP. In the comparison of MP as control (Fig. 2b), the results suggest that pollen mother cells had reached or been close to meiosis, and beginning to retain material and energy for subsequent pollen maturation [8]. In the comparison between MRP and PM (Fig. 2c), the results implied that in later stage of pollen development, beside continuing energy substance reserving, microspores also provided the basis for the subsequent pollination and fertilization of reserve materials [5, 8, 42, 43]. Flavonoids in PM stage are not only the components of pollen coat but also free radical scavengers, therefore, the up-regulation of flavonoids biosynthesis could be contributive to prevent or reduce pollen’s suffering the adversity environment in the pollination process, especially the abiotic stresses [44]. Phosphorylation signals contribute to the identification of pollen grains and stigmas [42]. The axon guidance allows the pollen tube to grow in the right direction, guiding the pollen tube to the location of the pearl hole so that the fertilization process can be successfully completed [8, 43].

### Different gene expression pattern during anther development

The DEGs expression analysis showed that the genes which enriched in pathways like cell cycle, homologous recombination and meiosis, were down-regulated (Fig. 3b, c, d). In the SCP, these genes were the highest expression and reached the lowest in the PM, and the expression trend gradually decreased along with the process of anther development, which indicated that the three pathways played a decisive role in early stages of anther development and the importance of regulation gradually weakened along with the anther development [1, 45]. However, the expression of genes which were enriched in starch and sucrose metabolism and carbohydrate digestion and absorption pathways increased gradually along with the process of anther development (Fig. 3e and Additional file 5: Figure S2F), which indicated that in later phases of anther development the energy metabolism process was mainly carried out in the microsporocyte [5, 46].

There was no consistent expression profiling in the DEGs which enriched in plant hormone signal transduction and carbon metabolism pathways (Fig. 3f and Additional file 5: Figure S2E). The expression of auxin synthesis gene *AUX1* (CotAD_18928) slowly increased from SCP to MP, and rapidly increased in the MRP, and then quickly decreased in the PM (Fig. 6). The expression profiling indicated that *AUX1* played a crucial role in the MRP [9]. However, the abscisic acid synthesis gene *PYL* (CotAD_22336) showed stably up-regulated expression during the whole anther development (Fig. 6). These different expression patterns indicated that the *PYL* might play an important but different role with *AUX1* during the whole anther development [47, 48].

### Meiosis as the most important stage in pollen development process

The expression pattern analysis found that cluster 8 had enriched the most DEGs, followed by cluster 4 and cluster 1 in turn (Fig. 4a and Additional file 6: Table S4). Cluster 8 and cluster 4 were in the meiosis stage, where the DEGs reached the highest, indicating that a large number of specific genes were involved in MP and the complexity of gene regulation was existedduring meiosis. Therefore, it could be considered that MP was the most important stage in the whole anther development period [35]. In cluster 1, DEGs reached to the highest in the SCP, which indicating that the proliferation of pollen mother cells also requires a large number of different genes to participate in. SCP as an early stage of MP, a lot of specific genes are speculated to begin to be expressed in pollen mother cells, which reserved material and energy for the following meiosis to ensure the smooth progress of meiosis and promote the pollen development process [1]. This conjecture can be confirmed from the SCP as control and compared with the MP, MRP and PM, respectively, the DEGs mostly clustered in the pathways related to maturation and meiosis of oocytes.

GO enrichment analysis revealed that DEGs in cluster 8 were mainly enriched in oxidation-reduction process, transport, membrane, carbohydrate metabolic process and ATP binding terms (Fig. 4c). Presumably, pollen mother cells were required to obtain a lot of substances to satisfy the meiosis process. Such course is an intensive ATP consuming and reactive oxygen species (ROS)-producing process causing pollen mother cells to mobilize the active oxygen scavenging mechanism to prevent damaged by oxidation. In cluster 4, a part of DEGs enriched in the hydrolase activity and protein binding terms (Fig. 4c), which regulate the degradation of spindles and cytoskeletons restructuring. The DEGs GO enrichment in cluster 1 revealed that the DEGs were mainly enriched in terms of DNA binding, nucleosome assembly and DNA replication, which was consistent with the proliferation of pollen mother cells in the SCP through mitosis (Fig. 4c).

### miRNA involved in regulation of anther development

MiRNA regulation of the target gene was mainly achieved by complementary pairing with the protein-coding region of the target gene and cutting the target gene or suppressing the translation of the target [49].

**Fig. 8:**
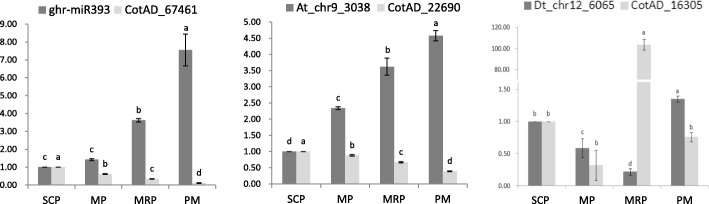
Real-time quantitative analysis of miRNAs and target genes. qRT-PCR analysis of the expression of miRNAs (ghr-miR393, At_chr9_3038 and Dt_chr12_6065) and their corresponding target genes (CotAD_67461, CotAD_22690 and CotAD_16305). Significant differences between treatments are indicated by letters above each bar (*P ≤ 0.05*). Data are present as means ± SE (*n* = 5)

*MPS1* (CotAD_67461), which regulated cell cycle function during anther development, was regulated by ghr-miR393 at gene expression. The *MPS1* showed a sustained up-regulated expression and the ghr-miR393 showed an opposite trend (Fig. 8). Sequence alignment analysis in miRBase found that there was one base difference between the sequence of the ath-miR393 and ghr-miR393. In *Arabidopsis thaliana*, *mTIR1* could promote the expression of ath-miR393, resulting in flowering delay [50]. The same trend was found between miRNA Dt_chr12_6065 and the target gene *PME40* (CotAD_16305), this gene was associated with starch and sucrose metabolism (Fig. 8). Auxin is necessary for anther development. At different developmental stages, anther has strict requirements on auxin concentration, high or low auxin concentrations may lead to abnormal development of anthers. Studies have shown that too low concentrations of auxin can lead to male sterility [51], while excessive concentrations of auxin can cause anther indehiscence, which affecting the pollination and fertilization process [31].

Auxin signaling primarily depended on four partially redundant F-box proteins of the TIR1/AFB2 auxin receptor (TAAR) clade to trigger the degradation of AUX/IAA transcriptional repressors [52]. *TIR1* is the target gene of At_chr9_3038 (the sequence of At_chr9_3038 is same as the ath-miR393), the expression of At_chr9_3038 increased gradually with the anther development process and the opposite expression profile of *TIR1* (Fig. 8) suggesting that in the early stages of anther development, a higher concentration of auxin was required, and the auxin concentration decreased gradually with the anther development [51].

*IAA14* inhibits the expression of auxin responsive genes [31]. In our study, the expression profiling of *IAA14* was similar to that of At_chr9_3038 (Fig. 6), showing a gradual increase to inhibit the function of auxin in the later stages of anther development. It is speculated that during the anther development, a variety of ways of coordinated regulation were adopted to regulate the concentration of auxin, in order to reach the appropriate concentration of auxin in different stages of anther development [31, 53, 54].

Because of the stable and conserved characteristics of miRNAs, three miRNAs which could be used for the judgment of anther development stage according to the expression relationship were screened and confirmed by real-time quantitative RT-PCR. With the development of cotton anther, the order of expression amount is ghr-miR396a > Dt_chr7_8575 > ghr-miR2948-5p in SCP, Dt_chr7_8575 > ghr-miR396a > ghr-miR2948-5p in MP, ghr-miR2948-5p > Dt_chr7_8575 > ghr-miR396a in MRP, and Dt_chr7_8575 > ghr-miR2948-5p > ghr-miR396a in PM (Additional file 12: Figure S5). The relative expression amount of the three miRNAs provides a new method for the stages division of cotton anthers, and also provides a molecular basis at different period, but this reason and mechanism need to be further investigated.

## Conclusions

The results of this study reveal that different genes are involved in the regulation of anthers at different developmental stages and at the same time promote the development of anthers. The gene expression profile analysis suggests that the importance of meiosis in the entire anther development, this conclusion can explain the MP is most susceptible to environmental influences, resulting in the occurrence of male sterility. Through the prediction of miRNA target genes, it was found that miRNAs participate in the regulation of anther development. Especially in the precise regulation of plant hormone auxin during the process of cotton anther development, miRNAs play a very important role. The results will enhance our understanding of the genes expression and regulation during cotton anther development and would be valuable for clarifying the mechanism of plant anther development in response to internal and external environments.

## Methods

### Plant materials

The genetically standard line of upland cotton was planted in experimental field of Hunan Agricultural University [55]. According to our earlier studies [56], the sporogenuous cell proliferation (SCP), meiotic phase (MP), microspore release period (MRP) and pollen maturity (PM) four stages buds were harvested, respectively, in the morning at the peak flowering period. Anthers collected from buds at four stages and then stored at freezer used to the transcriptomic and small RNA sequencing. The bud, stamen and anther size at four stages showed in Additional file 13: Figures S6A, S6B and S6C, and the each stage of cellular state were confirmed under optical microscope (Additional file 13: Figure S6D).

### RNA extraction and sequencing

Twenty buds were collected for each period to separate anther tissue. Approximately 20 μg of total RNA from each sample was extracted from isolated anthers using TRIzol reagent kit (Invitrogen, Carlsbad, CA, US) according to the manufacturer’s specification. The yield of RNA was determined using a NanoDrop 2100 spectrophotometer (Thermo Scientific, USA), and the integrity was evaluated using agarose gel electrophoresis stained with ethidium bromide. The mRNA were enriched by magnetic beads coated with Oligo (dT), and then randomly fragmented by ultrasound. Using the mRNA fragments, first and second strand cDNAs were synthesized with six-base primers. Double stranded cDNAs were repaired and connected to sequencing heads. The total RNA was subjected to gel electrophoresis; fragments with a certain size were recovered and 3’and 5′-end linkers were added; the platform of Illumina HiSeq 2500 was adopted to have small RNA sequencing (transcriptome and small RNA sequencing were both performed by OE biotech company (Shanghai). The sequencing data are deposited in NCBI Sequence Read Archive (SRA, http://www.ncbi.nlm.nih.gov/Traces/sra) with accession number SRP143909.

### Data analysis

Two biological repetitions were taken at each stage, respectively named as TC1–1 and TC1–2(SCP), TC2–1 and TC2–2 (MP), TC3–1 and TC3–2(MRP), TC4–1 and TC4–2(PM). The reference genome and transcriptome link of upland cotton were ftp://public.genomics.org.cn/BGI/cotton/Gossypium_hirsutum/Gossypium_hirsutum_v1.0.gz. The comparative analysis of cotton reference genome is made for clean bases [57]. Gene expression was calculated and subsequently normalized to RPKM [58]. The uniform screening conditions for differential mRNA and differential microRNA (miRNA) were *p* < =0.05, and fold change > 2 or fold change < 0.5. The Bowtie software is adopted to compare the sequence obtained by sequencing with the miRNA mature body sequence in miRBase, which is considered to be a known miRNA [59]. Combined with homologous miRNA sequence of cotton and the RNA secondary structure prediction software like RNA fold, the miRDeep2 software is adopted to predict new mature body. The screened differentially expressed genes were made GO and KEGG analyses [60]. The K-means clustering algorithm was adopted to analyze the expression pattern, and the on-line prediction website (http://plantgrn.noble.org/psRNATarget/) was used to perform the target gene prediction, and the prediction parameters: Maximum Entropy = 4.0; Target accessibility = 50. The String website (https: //string. Embl.de/) was used to predict protein interactions.

### Real-time quantitative RT-PCR

The residual RNA of sequencing was used for real-time quantitative RT-PCR. The primer pairs (Additional file 14: Table S8) used for real-time quantitative RT-PCR were designed using Roche LCPDS2 software and synthesized by Generay Biotech (Generay, PRC).

The mRNAs real-time quantitative RT-PCR method was according to previous studies [61].The microRNAs quantification was performed with a two-step reaction process: reverse transcription (RT) and PCR. Each RT reaction consisted of 0.5 μg RNA, 2 μl of miScript HiSpec Buffer, 1 μl of Nucleics Mix and 0.5 μl of miScript Reverse Transcriptase Mix (Qiagen, Germany), in a total volume of 10 μl. Reactions were performed in a GeneAmp® PCR System 9700 (Applied Biosystems, USA) for 60 min at 37 °C, followed by heat inactivation of RT for 5 min at 95 °C. The 10 μl RT reaction mix was then diluted × 10 in nuclease-free water and held at − 20 °C. Real-time PCR was performed using LightCycler® 480 II Real-time PCR Instrument (Roche, Swiss) with 10 μl PCR reaction mixture that included 1 μl of cDNA, 5 μl of 2 × QuantiFast® SYBR® Green PCR Master Mix (Qiagen, Germany), 0.2 μl of universal primer (Qiagen, Germany), 0.2 μl of microRNA-specific primer and 3.6 μl of nuclease-free water. Reactions were incubated in a 384-well optical plate (Roche, Swiss) at 95 °C for 5 min, followed by 40 cycles of 95 °C for 10 s, 60 °C for 30 s. Each sample was run in triplicate for analysis. At the end of the PCR cycles, melting curve analysis was performed to validate the specific generation of the expected PCR product.

The expression levels of mRNAs and miRNAs were normalized to *Actin* (NM_001327051.1) and *5.8S* gene, respectively. The expressin amounts were calculated using the 2^-ΔΔCt^ methd [62].

## Additional files

Additional file 1: **Table S1.** Data statistics for transcriptome sequencing. (XLSX 11 kb)

Additional file 2: **Figure S1.** Sample-to-Sample clustering. (PDF 9 kb)

Additional file 3: **Table S2.** Differentially expressed genes KEGG enriched TOP10. (XLSX 21 kb)

Additional file 4: **Table S3.** Metabolism and signaling pathway gene expression levels. (XLSX 13 kb)

Additional file 5: **Figure S2.** The expression profiles of gene related with metabolism and signal pathways during anther development. (PDF 221 kb)

Additional file 6: **Table S4.** Differential genes expression. (XLSX 31 kb)

Additional file 7: **Table S5.** Data statistics for microRNA sequencing. (XLSX 11 kb)

Additional file 8: **Table S6.** Target gene prediction for differentially expressed miRNAs, which between SCP and MRP, and KEGG enrichment of target genes. (XLSX 11 kb)

Additional file 9: **Table S7.** Prediction of target genes for differentially expressed miRNAs, which between SCP and PM and KEGG enrichment of target genes. (XLSX 11 kb)

Additional file 10: **Figure S3.** Target gene enrichment and cluster analysis. A: KEGG enrichment of miRNAs, differentially expressed between SCP and MRP, target genes; B: Expression profiles of miRNAs target genes; C: GO enrichment of target genes (BP: Biological process; CC: Cellular component; MF: Molecular function). (PDF 429 kb)

Additional file 11: **Figure S4.** Target gene enrichment and cluster analysis. A: KEGG enrichment of miRNAs, differentially expressed between SCP and PM, target genes; B: Expression profiles of miRNAs target genes. C: GO enrichment of target genes (BP: Biological process; CC: Cellular component; MF: Molecular function). (PDF 501 kb)

Additional file 12: **Figure S5.** Three miRNAs were used for the judgment of anther development stage according to the expression relationship were confirmed by real-time quantitative RT-PCR. The order of expression amount is ghr-miR396a > Dt_chr7_8575 > ghr-miR2948-5p in SCP, Dt_chr7_8575 > ghr-miR396a > ghr-miR2948-5p in MP, ghr-miR2948-5p > Dt_chr7_8575 > ghr-miR396a in MRP, and Dt_chr7_8575 > ghr-miR2948-5p > ghr-miR396a in PM. (PDF 67 kb)

Additional file 13: **Figure S6.** The four stages of cotton pollen development. Cotton bud and anther sizes at the sporogenuous cell proliferation (SCP), meiotic phase (MP), microspore release period (MRP) and pollen maturity (PM) four continuous stages. A: Bud; B: Stamen; C: Anther; D: Pollen cellular state under optical microscope (stained with acetocarmine). (PDF 165 kb)

Additional file 14: **Table S8.** Primers list in real-time qRT-PCR. (XLSX 11 kb)

## Abbreviations

DEG: Differentially expressed gene
GO: Gene ontology
KEGG: Kyoto encyclopedia of genes and genomes
MP: Meiotic phase
MRP: Microspore release period
PM: Pollen maturity
ROS: Reactive oxygen species
SCP: Sporogenuous cell proliferation
