# Supplementary figures and images for: Insights into the cotton anther development through association analysis of transcriptomic and small RNA sequencing
Source: BMC Plant Biol. 2018 Aug 3;18:154. doi: 10.1186/s12870-018-1376-4 (PMC6091077; doi:10.1186/s12870-018-1376-4)

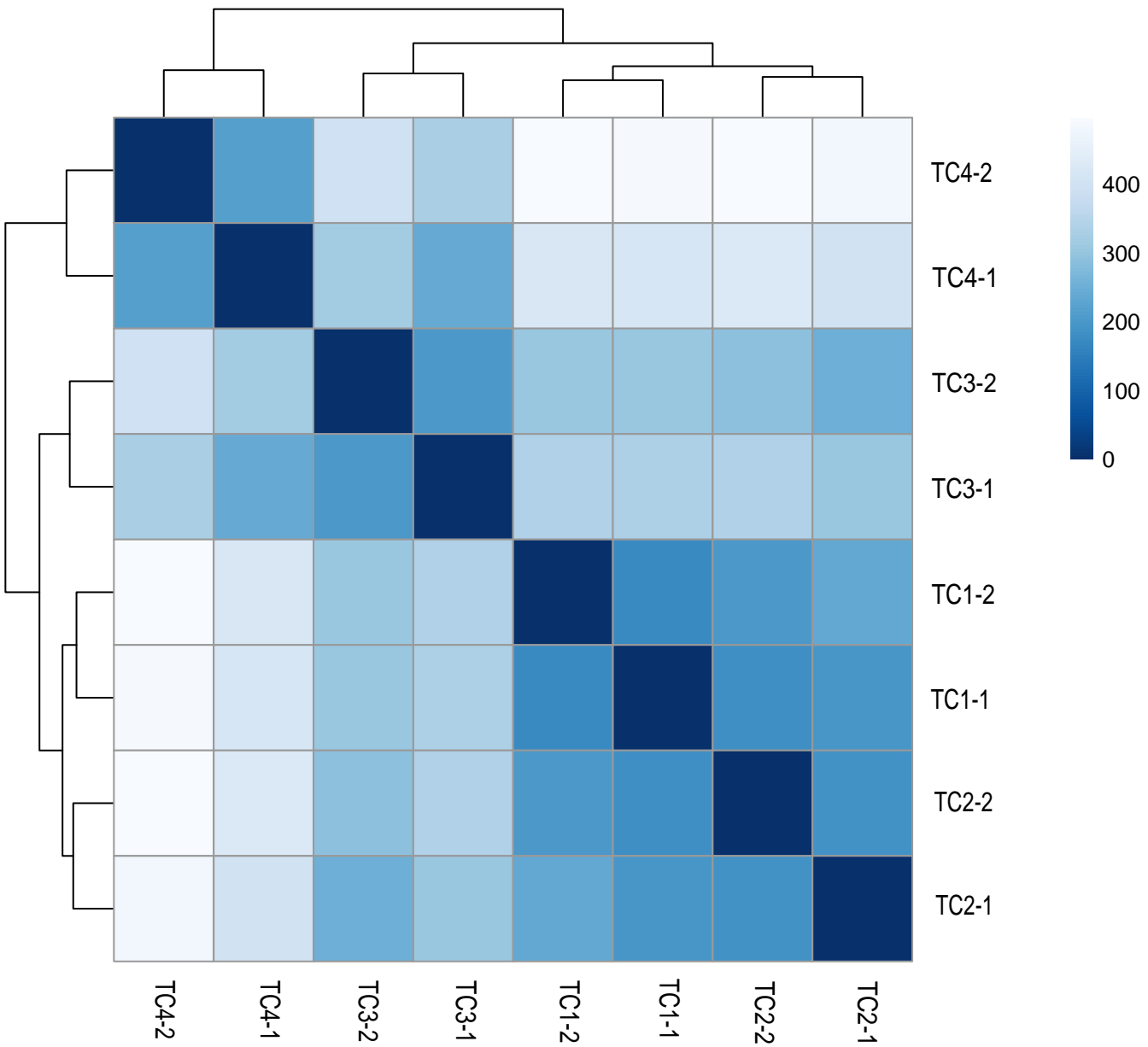

Supplement: Supplementary file 2 — Figure S1. Sample-to-Sample clustering. (PDF 9 kb) [file 12870_2018_1376_MOESM2_ESM.pdf]

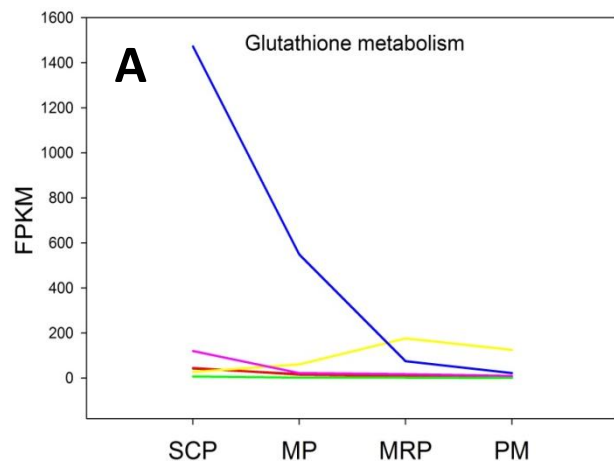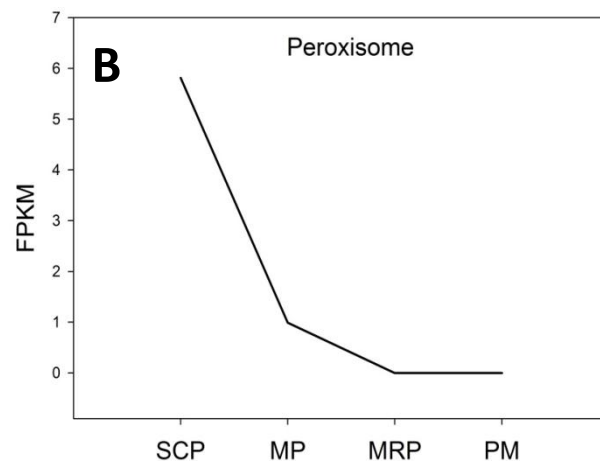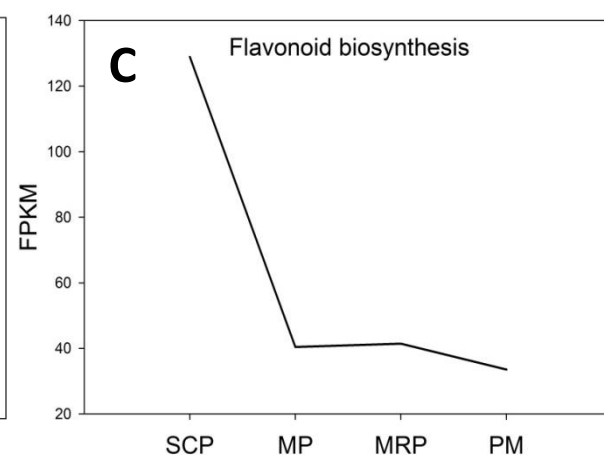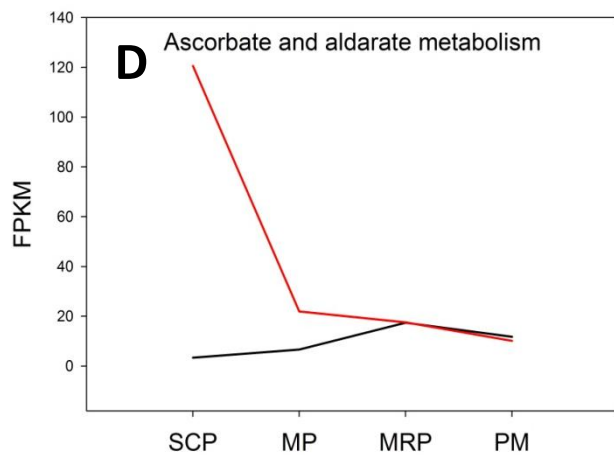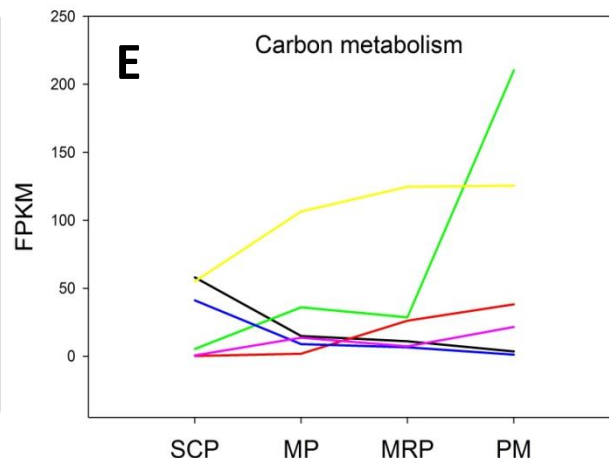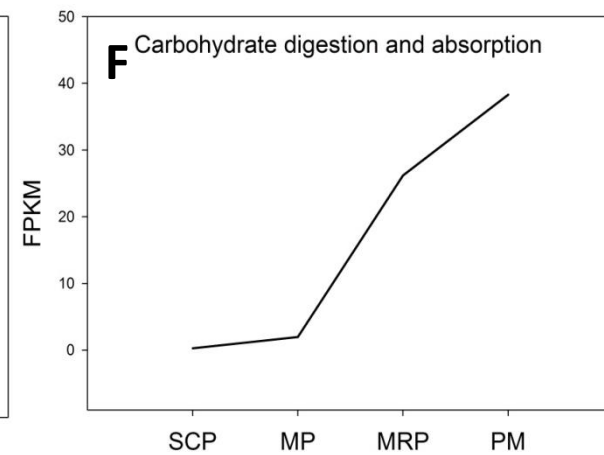

Supplement: Supplementary file 5 — Figure S2. The expression profiles of gene related with metabolism and signal pathways during anther development. (PDF 221 kb) [file 12870_2018_1376_MOESM5_ESM.pdf]

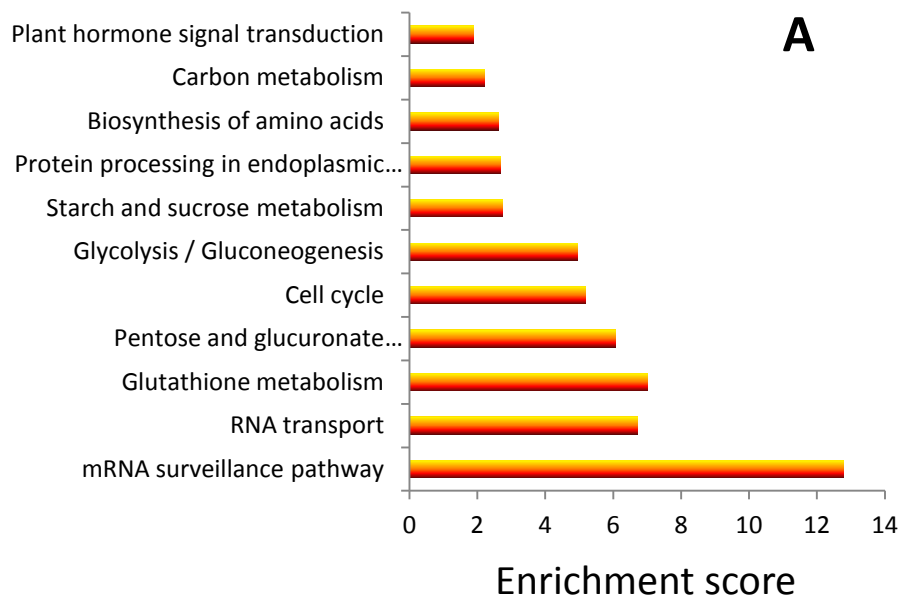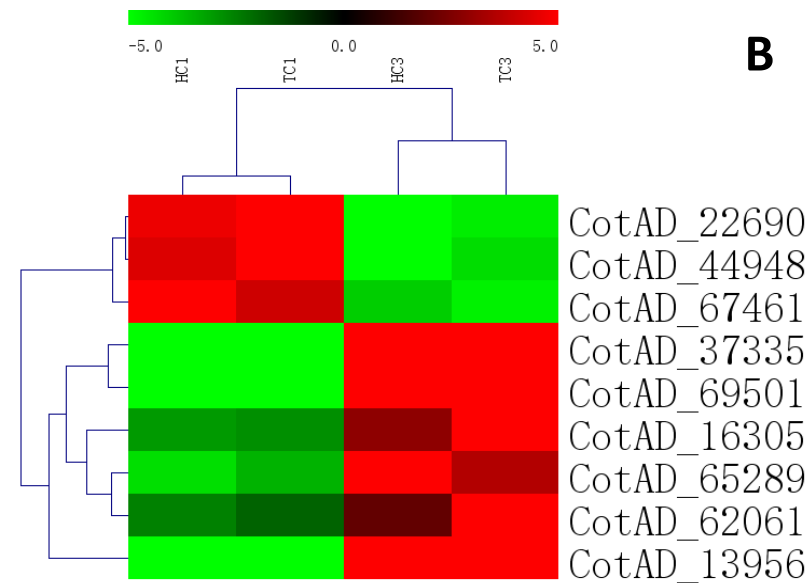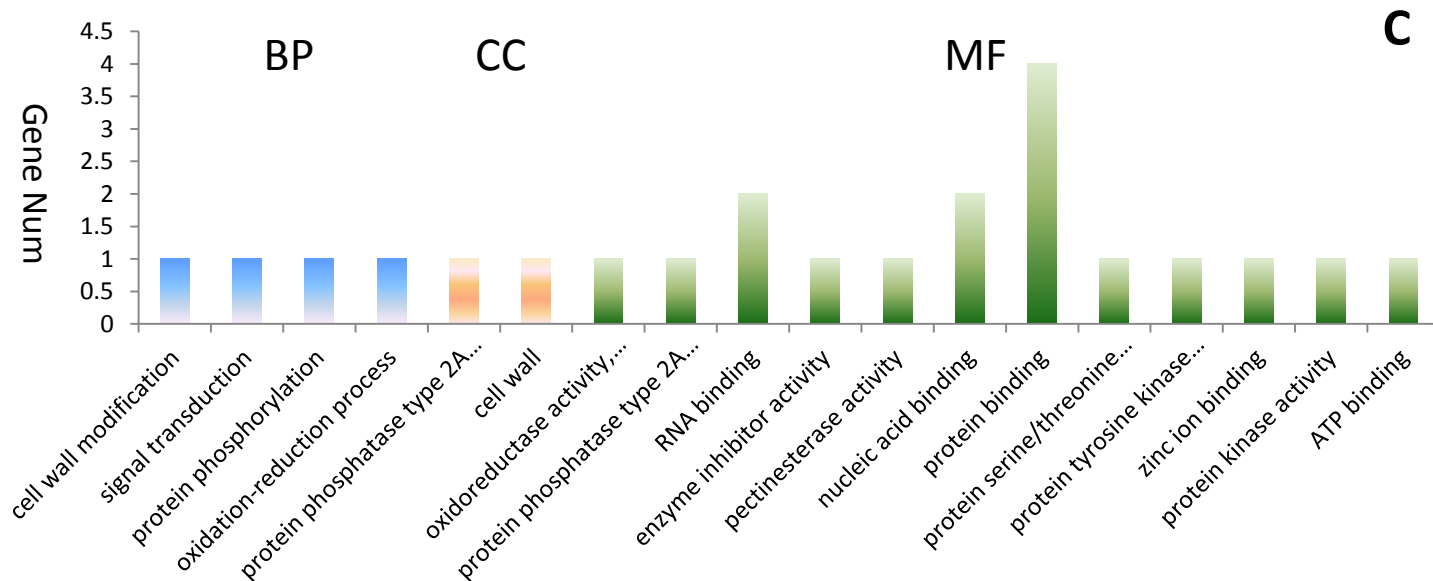

Supplement: Supplementary file 10 — Figure S3. Target gene enrichment and cluster analysis. A: KEGG enrichment of miRNAs, differentially expressed between SCP and MRP, target genes; B: Expression profiles of miRNAs target genes; C: GO enrichment of target genes (BP: Biological process; CC: Cellular component; MF: Molecular function). (PDF 429 kb) [file 12870_2018_1376_MOESM10_ESM.pdf]

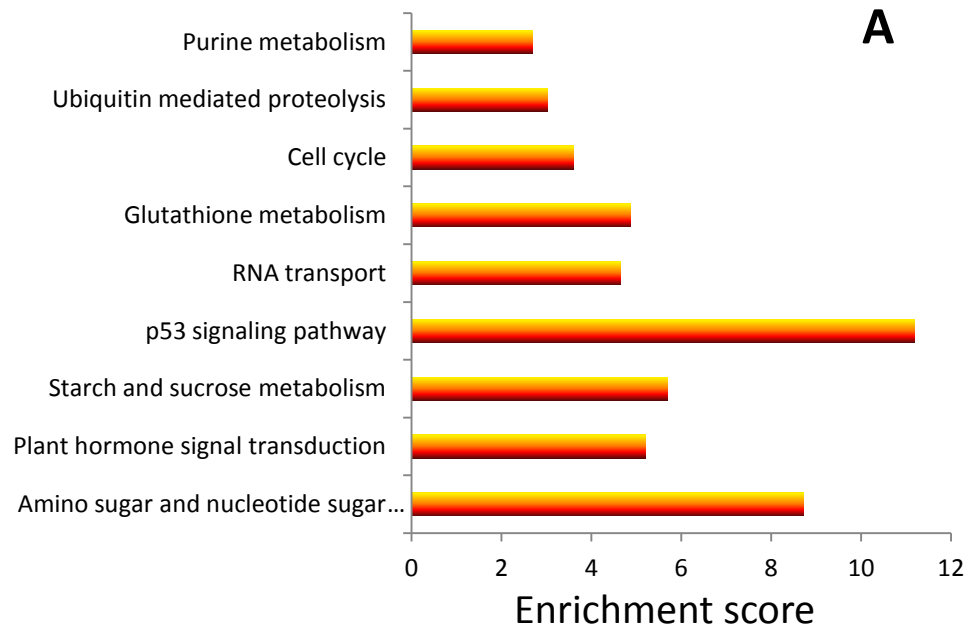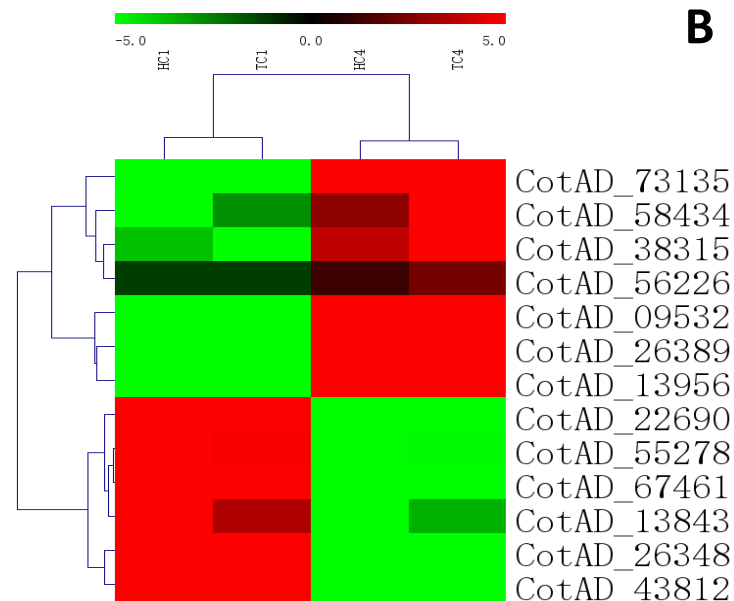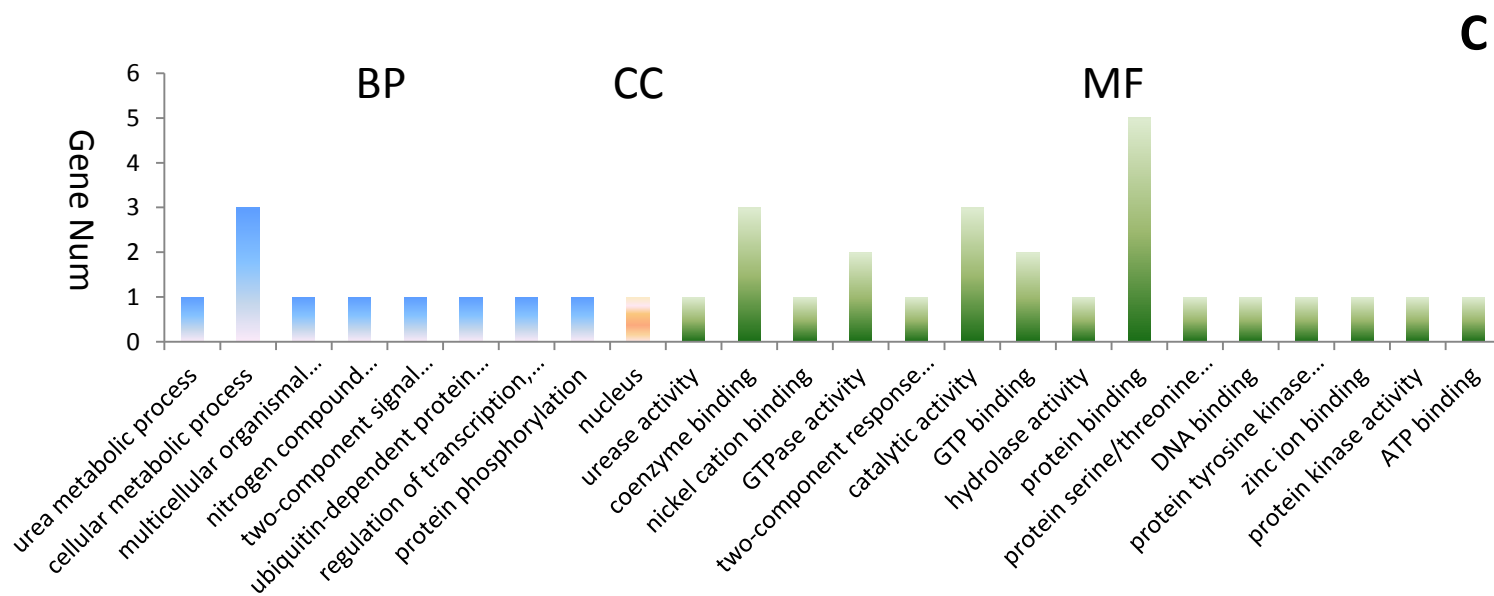

Supplement: Supplementary file 11 — Figure S4. Target gene enrichment and cluster analysis. A: KEGG enrichment of miRNAs, differentially expressed between SCP and PM, target genes; B: Expression profiles of miRNAs target genes. C: GO enrichment of target genes (BP: Biological process; CC: Cellular component; MF: Molecular function). (PDF 501 kb) [file 12870_2018_1376_MOESM11_ESM.pdf]

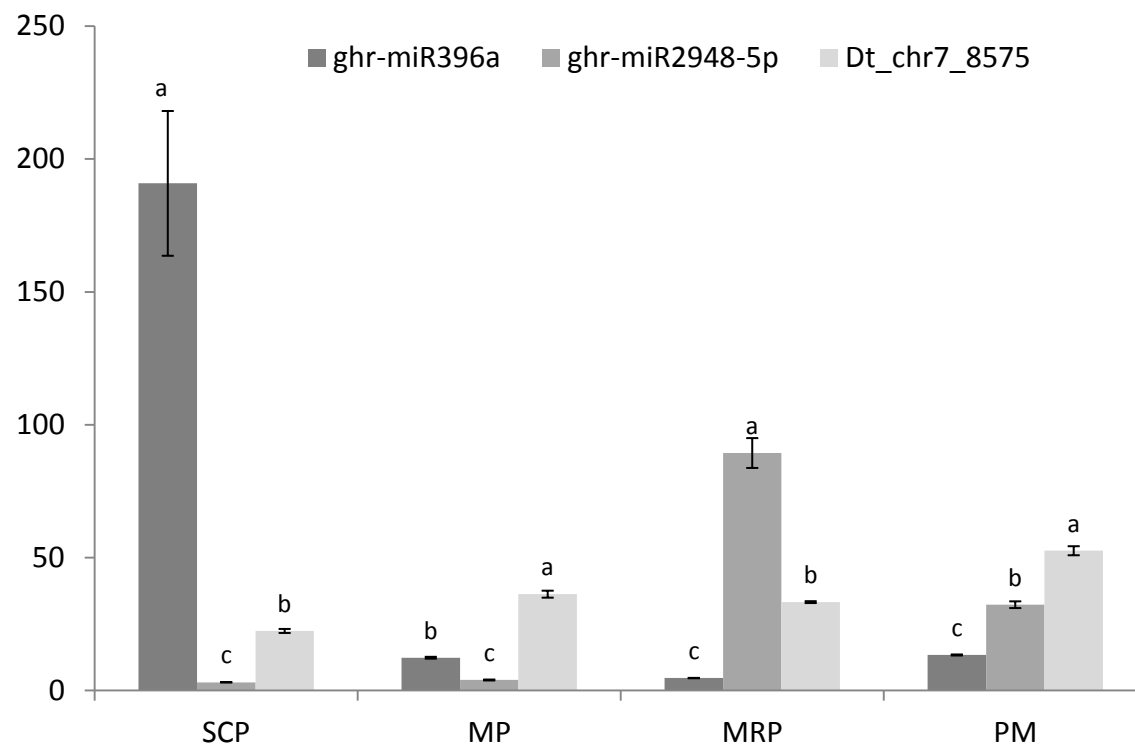

Supplement: Supplementary file 12 — Figure S5. Three miRNAs were used for the judgment of anther development stage according to the expression relationship were confirmed by real-time quantitative RT-PCR. The order of expression amount is ghr-miR396a > Dt_chr7_8575 > ghr-miR2948-5p in SCP, Dt_chr7_8575 > ghr-miR396a > ghr-miR2948-5p in MP, ghr-miR2948-5p > Dt_chr7_8575 > ghr-miR396a in MRP, and Dt_chr7_8575 > ghr-miR2948-5p > ghr-miR396a in PM. (PDF 67 kb) [file 12870_2018_1376_MOESM12_ESM.pdf]

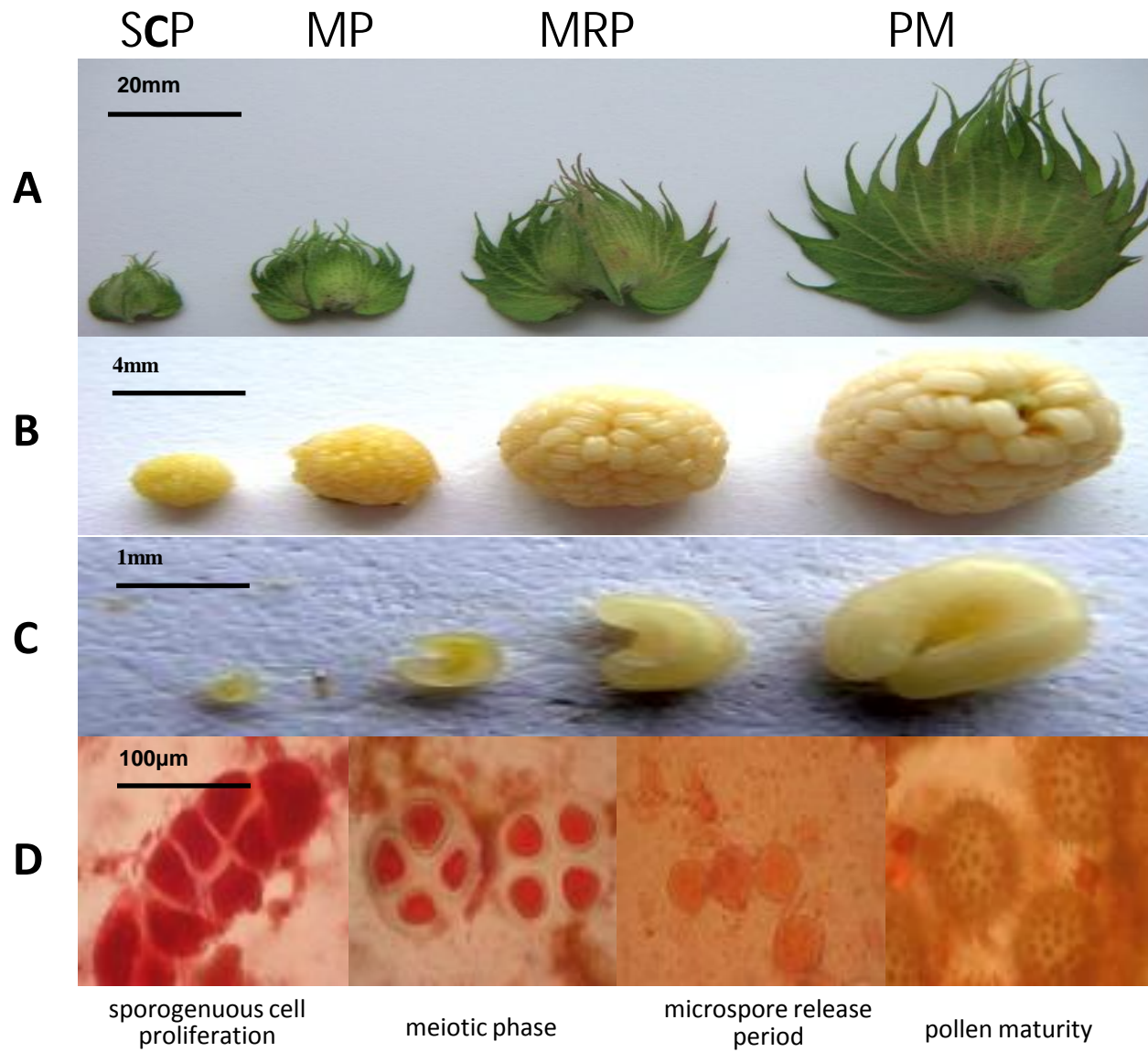

Supplement: Supplementary file 13 — Figure S6. The four stages of cotton pollen development. Cotton bud and anther sizes at the sporogenuous cell proliferation (SCP), meiotic phase (MP), microspore release period (MRP) and pollen maturity (PM) four continuous stages. A: Bud; B: Stamen; C: Anther; D: Pollen cellular state under optical microscope (stained with acetocarmine). (PDF 165 kb) [file 12870_2018_1376_MOESM13_ESM.pdf]
